# Chronic bilateral heel pain in a child with Sever disease: case report and review of literature

**DOI:** 10.1186/1757-1626-2-9365

**Published:** 2009-12-21

**Authors:** Fred C Sitati, John Kingori

**Affiliations:** 1 Orthopedic Unit, PCEA Kikuyu Hospital, Kikuyu, Kenya

## Abstract

We are presenting a case report of a 10-year-old male with a 1 year history of bilateral heel pain. Sever disease is self limiting condition of calcaneal apophysis. It is the most common cause of heel pain in the growing child. There is no documented case of this condition in this region. This case highlights the clinical features of this self limiting disorder as seen in this patient and reviews the current literature.

## Case presentation

A 10 year-old male Kenyan Bantu presented in our clinic with a 1 year history of bilateral heel pain. It had worsened over a two week period and at times he walked on his toes. The pain was of spontaneous onset with no history of trauma. The pain was aggravated by activity and relieved by rest. It was dull in nature and non-radiating. There was no associated history of weight loss, fever or anorexia. There was no past medical history of chronic illness, allergy, hospital admission or surgery. There was no history of similar disease in the family.

Examination revealed a well-nourished boy, not pale, normal skin, normal vital signs. He walked with a limping gait. Examination of the foot revealed marked tenderness at the posterior calcaneus more on the right side.

Investigations done included,

• Complete blood cell count-Found normal

• Erythrocyte sedimentation rate- 16 mm/hr (normal <20 mm/hr)

• Alkaline phosphatase- 95 U/l (normal <270 units/l)

• Serum calcium-9.4 mg/dl (normal 8.1-10.4 mg/dl)

• Rheumatoid factor- negative

**Figure 1:**
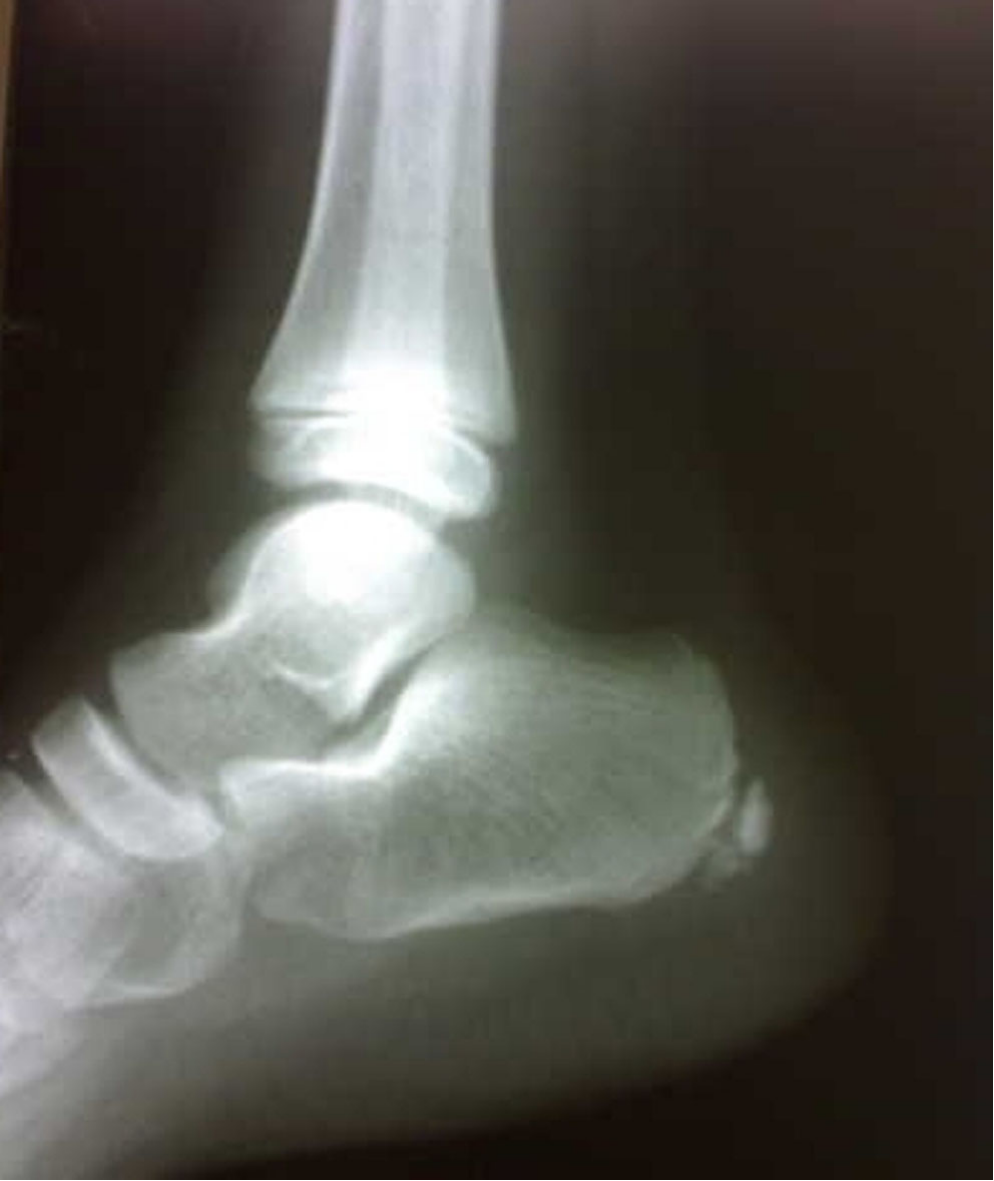
**Lateral radiograph of the right foot**. Note the sclerosis and fragmentation of the calcaneal apophysis.

Radiograph revealed sclerosis and fragmentation within the calcaneal apophysis (Figure 1). A diagnosis of Sever disease was made and the patient was advised to stop activities that cause the pain such as sports and was prescribed oral ibuprofen 400 mg three times a day for three weeks and diclofenac gel to rub at the heel. The pain had resolved within two months of instituting treatment and the patient returned to sports activities.

## Discussion

Sever disease also known as calcaneal apophysitis is a common problem in the western world in growing children. There are no reported figures on its occurrence globally. There are no documented cases of this condition in African literature. We consequently present this case found in our region to highlight the features of this disease.

Sever disease was first described in 1912, by James Warren Sever, as an inflammatory injury to the calcaneal apophysis associated with muscle strain in the immature skeleton [1], It is a nonarticular osteochondrosis of the calcaneal apophysis. It is similar to Osgood-Schlatter disease in the knee and little-leaguer's elbow in the elbow [2]. The inflammatory process is attributed to decreased resistance to shear stress at the bone-growth plate interface [3]. Siffert believed that repeated microtrauma caused mechanical disruption [4].

Sever disease is the most common cause of heel pain in the growing child. The average age at presentation is 11 years, ranging from 8 to 15 years [3]. In an audit of youth soccer injuries, Sever disease was found to be most common during the beginning of a pubertal growth spurt [5]. A boy-to-girl ratio is 2-3:1. The incidence of bilaterality is approximately 60% [3]. The classical case is usually a preadolescent boy with chronic heel pain. Pain is increased with activity. There no constitutional symptoms. Examination usually reveals no visible abnormalities apart from pain with medial-to-lateral compression of the posterior part of the calcaneus [6]. Toe walking usually relieves pain. The patient may have slightly decreased dorsiflexion due to minimal heel cord tightness. The diagnosis is made purely upon clinical examination [4].

Plain radiographs do not reveal characteristic features of Sever disease. Ossification irregularities with sclerosis and fragmentation within the apophysis are normal features of a developing calcaneus. The lateral calcaneal view and axial (Harris) view are taken only when the history is not classic, when the patient has night pain, swelling, exquisite tenderness, or when a diagnosis other than Sever disease is suspected [7]. A bone scan may be helpful when a stress fracture is suspected [6]. An MRI can show areas of bone edema and hemorrhage, or a bone bruise, within the metaphyseal bone of the calcaneus [8].

The laboratory investigations in our case were normal as expected in this condition [9]. The treatment of this disease is non operative [6]. Various modalities have been used including rest, anti-inflammatory medications (NSAIDs), splints and casts. Parents must be reassured that the condition is self limiting and surgery is not an option as a quick remedy. Most cases resolve within months of treatment [7].

In a child with heel pain, the differential diagnosis may include Achilles tendonitis, retrocalcaneal bursitis, calcaneal stress fractures, calcaneal cysts, osteomyelitis, and plantar fasciitis [7,8]. Usually, these causes can be ruled out with a well-performed clinical evaluation.

## Conclusion

Sever disease is a common condition in the growing child. We present the clinical features of one such case found in our region.

## Abbreviations

NSAIDs: Non Steroidal anti Inflammatory Drugs; MRI: Magnetic Resonance Imaging.

## Competing interests

The authors declare that they have no competing interests.

## Authors' contributions

FCS, Searching the literature; Major contributor in writing the manuscript. Writing the case report and discussion section. JK, Searching the literature. Contributor in writing the case report and discussion section. All authors read and approved the manuscript

## Consent

Written informed consent was obtained from the patient for publication of this case report and accompanying images. A copy of the written consent is available for review by the journal's Editor-in-Chief.

## References

[B1] Sever JW Apophysitis of the os calcis NY Med J 1912 95 1025

[B2] Cassas KJ Cassettari-Wayhs A Childhood and adolescent sports-related overuse injuries Am Fam Physician 2006 73 6 1014 1022 16570735

[B3] Micheli LJ Ireland ML Prevention and management of calcaneal apophysitis in children: An overuse syndrome J Pediatr Orthop 1987 7 34 38 3793908 10.1097/01241398-198701000-00007

[B4] Siffert RS The osteochondroses Clin Orthop Relat Res 1981 158 2 3 7273518

[B5] Price RJ Hawkins RD Hulse MA Hodson A The Football Association medical research programme: An audit of injuries in academy youth football Br J Sports Med 2004 38 466 471 10.1136/bjsm.2003.005165 15273188 PMC1724880

[B6] Ishikawa SN Conditions of the calcaneus in skeletally immature patients Foot Ankle Clin 2005 10 503 513 10.1016/j.fcl.2005.03.001 16081017

[B7] Morrissy RT Weinstein SL Lovell and Winter's Pediatric Orthopedics 2001 5 Philadelphia: Lippincott Williams & Wilkins 1206

[B8] Ogden JA Ganey TM Hill JD Jaakkola JI Sever's injury: A stress fracture of the immature calcaneal metaphysis J Pediatr Orthop 2004 24 488 492 15308897 10.1097/00004694-200409000-00007

[B9] Weber JM Vidt LG Gehl RS Montgomery T Calcaneal stress fractures Clin Podiatr Med Surg North Am 2005 22 45 54 10.1016/j.cpm.2004.08.004 15555842

